# The Claims of Hospitals

**Published:** 1888-09-08

**Authors:** R. Brudenell Carter

**Affiliations:** Ophthalmic Surgeon to St. George's Hospital and the National Hospital for the Paralysed and Epileptic; Being a Revised Edition of the Mansion House Address Delivered Two Years Ago.


### The Claims of Hospitals.

By R. Brudenell Carter, F.R.C.S., Ophthalmic Surgeon to St. George's Hospital and the National Hospital for the Paralysed and Epileptic; being a revised edition of the Mansion House Address delivered two years ago.

*(Continued from p. 352.)*

I cannot refrain from mentioning an example of the effects of discouraging legitimate experiment. In 1851, the year of the first Great Exhibition, I lived for some months in the London Hospital, doing the duty of the resident medical officer, who was disabled by illness. Until about that time, the disease called pneumonia, or inflammation of the lungs, had been customarily treated by bleeding from the arm, and it was not only attended by a very high rate of mortality, but also by very tedious recovery in the most favourable cases. Dr. Hughes Bennett, of Edinburgh, had successfully treated a few cases without bleeding; and I had discussed the reports of these cases with the physicians under whom I was acting. One afternoon, after the physician's visit, I admitted a man with acute pneumonia; and, from some reason founded on his state, and on the aforesaid discussion, refrained from bleeding him. The next day, when the physician came, he approved the course I had pursued, and bleeding was not practised. The man not only got well, but made what was then thought a very rapid recovery. We were never without pneumonia in the London Hospital, and we were encouraged to feel our way farther in the same direction. Our cases were carefully selected, and they did well. They were, I believe, the first treated in London without bleeding, and they attracted much attention. We had, that year, many foreign visitors; and one day I did the honours of the hospital to a physician from Florence, and took him to the bedsides of half-a-dozen patients, in various stages of pneumonia, none of whom had been bled, and who were all doing well. He expressed the greatest interest in them, and I said, "No doubt you will try this method yourself when you return home?" "No," he replied, "I cannot do that; for if a patient dies under my care, after I have done for him all that is customary, I have nothing to fear; but if it were shown that I had omitted to do something that was customary, I should be liable to civil damages at the suit of his relations."

I do not know what may be the present state of medicine in Florence, or whether the law mentioned by my visitor has been repealed or changed. But this I do know, that in England scarcely anyone is now bled for pneumonia, that the mortality from the disease is comparatively trifling, and that convalescence is seldom unduly prolonged. Such has been the ultimate result of a hospital experiment; and it is worth while to remember the conditions under which it was carried out. Until the principle was established, the patients were watched with the most unremitting care, both day and night, and all variations in their symptoms were noted with extreme solicitude, and with perfect preparedness to practise bleeding at any moment, if their condition had justified the belief that such a course would be beneficial. Sir James Paget once remarked to me that, when a new method of treatment was being put in practice at a hospital, the patients selected for it had always a better chance than others. Everyone concerned, from the head physician or surgeon to the dresser or the nurse, was so anxious that the new method should be tried fairly that, as a matter of fact, it was generally tried unfairly; the patients receiving, from everybody, something more than their share of watchfulness and attention. I do not know how this may be as a rule, but it was certainly true of our experiment in abstaining from bleeding. That experiment, because it was conducted in a hospital, in the presence of a large body of students soon to go forth into the world to apply the knowledge which they had acquired, and in the presence of the reporters of the medical journals, sufficed, in a few months, to modify the whole course of medical practice in England. The patients whose lives were saved by the experiment itself are scarcely worth remembering, in comparison with the amount of good which has been indirectly effected through their means.

And now, returning to my original contention, that hospitals are more valuable to the community on account of the knowledge which radiates from them than on account of the actual relief which is afforded within their walls, considerable as this may be, I think it follows that I cannot call any form of hospital maintenance efficient which does not adequately provide for the accomplishment of this radiation. In order so to provide it is necessary, first, that physicians and surgeons should be selected on some principle which affords a guarantee of their skill and character; and, secondly, that the hospital should be freely open and accessible as a place of instruction, either for students or for practitioners. Such conditions, it is manifest, can scarcely be completely fulfilled in any but what are called "general" hospitals; and the ideal arrangement of hospital accommodation for London would be to place a sufficient number of general hospitals at proper intervals of distance, and to attach to them as many "special" departments as the interests of medical science might from time to time require. The hospitals should be large enough to keep down excessive establishment charges, and numerous enough to prevent the sick from being called upon to travel long distances.

By way of contrast to this ideal arrangement, we have now in London eleven general hospital, with very imperfect arrangements for the opening or variation of special departments, and placed very irregularly, some of them too near together, and others too far apart; and we have seventy-one special hospitals—some large, some small, by which, whatever else may be done, establishment charges are almost indefinitely multiplied. These special hospitals may, I think, be classified under four heads. A very few of them, constituting what I will call the first class, are places in which professional knowledge is being actively advanced by capable workers, and in directions which appeal more to practitioners than to younger students. A second class consists of asylums; a third of mere survivals; and a fourth class are as much private establishments for procuring or improving business as if they were greengrocers' shops. Those which are asylums afford shelter and comparative comfort to many poor creatures suffering from chronic and generally incurable maladies; but they do little or nothing in the way of the advancement of medical science, and may be regarded as charities simply, limited in their influence to the actual inmates who are relieved. The survivals are a still more numerous class, which, once useful in a general sense, now affect only the actual patients, except in so far as they retard the progress of medical education. Foremost in this class I should place the six hospitals for diseases of the eye, with regard to which I feel disposed to take courage, and to say exactly what is on my mind. Five-and-thirty years ago, the diseases of the eye were not studied by practitioners generally, and the patients suffering from them were not received into general hospitals. I completed my studentship, and passed my examinations, without having ever seen a case of eye disease in hospital. At that time, if it had not been for the two special hospitals which then existed, persons suffering from eye disease would have remained unrelieved. At last a time came when this branch of surgery underwent development with great rapidity, and when attention became generally directed to its importance. A special eye department, with a special ophthalmic surgeon or surgeons, was before long attached to every great hospital, and the subject was systematically taught to students. The chief difficulty of the teachers, however, was to obtain patients. The poor had been so long accustomed to go to the eye hospitals that they continued the custom; and the six which now exist in London claim to have received, last year, no less than 2,644 in-patients and 50,422 out-patients.

These six eye hospitals have thirty-two surgeons and assistant surgeons, of whom all the better-known and more experienced men are also officers of general hospitals, and either are or might be employed in seeing eye cases in them. The net result, therefore, is that the patients of the special hospitals gain nothing, because they might be under the care of the same surgeons elsewhere; and 53,000 persons are annually withdrawn from the general hospitals, where their presence would be invaluable as affording abundant material for the purposes of instruction. The public would be great gainers, and nobody would lose, if all the six eye hospitals were closed, and if the money expended upon them, as well as their surgeons, were divided among the general hospitals. The effect would be that medical students generally would be far better taught in this branch of surgery than they can be at present, and that the chances of finding, in every country district, a doctor well qualified to treat the diseases of the eye would be very materially increased. Thousands of people who never go near a hospital, either general or special, would in the long run derive advantage from the change.

In saying that the patients of these survival special hospitals gain nothing, I perhaps somewhat understate the case, because nothing is so conducive to careful medical work as the presence and the questions of students. Where there are few or no students, and a great many out-patients, the work is very apt to degenerate into a routine, in which mistakes may easily be made as the results of weariness. Moreover, the presence of an adequate number of student assistants greatly facilitates many kinds of systematic examinations which would occupy too much time unless accomplished by the aid of division of labour.

With regard to my last class, the special hospitals which are simply private places of business, designed to impress the general public with the great skill and knowledge which Mr. Jones or Mr. Robinson brings to bear upon some particular kind of disease, or upon the diseases of some particular part or organ, it is not expedient that I should dwell. Very frequently the Mr. Jones or Mr. Robinson in question is not a person who would be elected to hospital office by the suffrages of his own profession; and, also frequently, he loves to surround himself by weak colleagues, who are not likely either to control his acts or to overshadow his reputation. The institutions themselves are often destitute of the most elementary requirements of efficiency, or even of safety, and the professional work is often badly done. Money which might be better expended elsewhere is withdrawn from really useful institutions; and, when the cases dealt with are of a kind which is not numerous, the centres of medical education are improperly deprived of the means of teaching. It would be impossible, generally speaking, for any non-medical person to decide what so-called hospitals might fairly be included within this last category, or to tell when an institution which had originally belonged to the first class was beginning to lapse into the state of mischievous survivalism.

It follows, if we must admit that the support of efficient hospitals is a matter of national concern, and not merely a matter of benevolence or almsgiving, that the public must be in want of guidance with regard to the directions in which their support should be extended. We have, in London, eighty-two real or so-called hospitals, most of them engaged, more or less, in a struggle for existence, and with every one of which the pecuniary and personal interests of many officials and servants are bound up. The tendency to survival does not appear to be in proportion to usefulness, but to be rather dependent upon the exertions and upon the qualities of the secretary and of the canvassers. It is certain that the money wasted by the multiplication of institutions, and by the machinery of appeals, would go a long way towards the supply of the real hospital needs of the metropolis. Is it possible, by any form of organisation, to control hospital development in such a way as to obtain the maximum of good from the minimum of expenditure?

An attempt in this direction was made, some years ago, by the Council of the Hospital Sunday Fund. I always disapproved of this attempt, which rested, in the first instance, upon a necessarily imperfect and misleading comparison between the expenditure of the several participating institutions; and at length I withdrew from the Council on account of a decision to withhold the grant from a particular hospital, on the score of certain alleged irregularities in its management. I thought then, and I think now, that the Council had not the means of discharging judicial functions, and that it was a mistake to take any step in the direction of entering upon them. The merits of the case in question seemed to me to be entirely irrelevant to this much larger issue.

There has, however, for some time past, been a prospect of legislation by which hospitals will be relieved from the burden of local rates; and, if any such measure were passed, hospitals would have to be defined. Might it not be possible, in pursuance of a suggestion which I made several years ago, and which has been made independently by Mr. Burford Rawlings, to establish a sort of Board, containing both medical and non-medical members, to which the claim of any institution to be so relieved might be submitted, and by which such claim should be adjudicated upon. Might not the law require a sanction or licence from such a body as a condition precedent to the acceptance of legacies? If institutions which had never been useful, or those which, having been useful, had ceased to be so, could in any way, however slightly, be thus ear-marked by the refusal of some privilege, it is probable that even those persons who were most disposed to support them would begin to look very critically into their pretensions.

There is yet one point more on which I would like to say a word, and it is the question of out-patients. We have all seen written, and heard spoken, a great deal of elegant English in condemnation of what has been called the "Out-patient System," and we have been told that out-patients are pauperised, and much more. All this reminds me of that book by Sir William Temple, which was described by Macaulay as the best ever written by any man upon the wrong side of a question of which he was entirely ignorant. Out-patients, and plenty of them, are absolutely indispensable for medical teaching; and if they failed to come willingly, it would be profitable to the community to pay them for coming. They consist of people who, at least apparently, are not very ill, and who, at all events, are not disabled. They enable physicians and surgeons to teach students to distinguish between trivial maladies and the beginnings of serious ones, and to treat both; and I need hardly say that a doctor is consulted about a trivial case much more often than about a grave one. If I were asked to define my ideal of a perfect family doctor, I should say it was furnished by the man whose habitual patients, coming to him about all small matters, were the least prone to severe diseases. A young practitioner would fail lamentably in his duties if he were able to recognize and to treat typhoid fever, or profound organic disease, and yet could neither recognise nor cure some slight and common ailment. For the attainment of the latter kind of knowledge, and, therefore, for the attainment of practical usefulness in daily life, the study of the maladies of out-patients is a matter of imperative necessity.

